# Exosomes From miR-19b-3p-Modified ADSCs Inhibit Ferroptosis in Intracerebral Hemorrhage Mice

**DOI:** 10.3389/fcell.2021.661317

**Published:** 2021-07-07

**Authors:** Xia Yi, Xiangqi Tang

**Affiliations:** Department of Neurology, The Second Xiangya Hospital, Central South University, Changsha, China

**Keywords:** intracerebral hemorrhage, adipose-derived stem cells, ferroptosis, miR-19b-3p, iron regulatory protein 2

## Abstract

**Objectives:** Effective treatments for intracerebral hemorrhage (ICH) are limited until now. Ferroptosis, a novel form of iron-dependent cell death, is implicated in neurodegeneration diseases. Here, we attempted to investigate the impact of exosomes from miR-19b-3p-modified adipose-derived stem cells (ADSCs) on ferroptosis in ICH.

**Methods:** Collagenase was used to induce a mouse model of ICH and hemin was used to induce ferroptosis in cultured neurons. Exosomes were isolated from mimic NC- or miR-19b-3p mimic-transfected ADSCs (ADSCs-MNC-Exos or ADSCs-19bM-Exos, respectively) and then administered to ICH mice or hemin-treated neurons. ICH damage was evaluated by assessing the neurological function of ICH mice and cell viability of neurons. Ferroptosis was evaluated in mouse brains or cultured neurons. The interaction between miR-19b-3p and iron regulatory protein 2 (IRP2) 3′-UTR was analyzed by performing luciferase reporter assay.

**Results:** Ferroptosis occurred in ICH mice, which also exhibited decreased miR-19b-3p and increased IRP2 expression. IRP2 was a direct target of miR-19b-3p, and IRP2 expression was repressed by ADSCs-19bM-Exos. Importantly, ADSCs-19bM-Exos effectively attenuated hemin-induced cell injury and ferroptosis. Moreover, ADSCs-19bM-Exos administration significantly improved neurologic function and inhibited ferroptosis in ICH mice.

**Conclusion:** Exosomes from miR-19b-3p-modified ADSCs inhibit ferroptosis in ICH mice.

## Introduction

Intracerebral hemorrhage (ICH), which is defined as the spontaneous extravasation of blood into the brain parenchyma, is a devastating kind of stroke with high disability and mortality rates (Zhang et al., 2013; Yu et al., 2019). Current knowledge of post-ICH neuronal death and related mechanisms is limited. Ferroptosis is a newly identified form of cell death that is different from apoptosis, necrosis and autophagy. Ferroptosis is characterized by iron-dependent lipid peroxide accumulation (Dixon et al., 2012). Accumulating researches have demonstrated that ferroptosis is involved in various cancers and neurodegenerative diseases (Zhi et al., 2017; Tang et al., 2018; Kenny et al., 2019; Weiland et al., 2019). Previous evidence suggests that inhibition of ferroptosis can achieve desirable therapeutic effects in neurodegeneration diseases including stroke (Tang et al., 2018). For instance, Alim et al. (2019) recently have demonstrated that pharmacological selenium supplementation blocks ferroptosis and improves behavior in a hemorrhagic stroke model.

Iron regulatory proteins (IRP1-2) are master regulators of cellular iron homeostasis (Wang et al., 2007). IRP2 (*IREB2*) has been shown to contribute to ferroptosis (Yao et al., 2019). Previous study has confirmed that IRP2 knockout mice exhibit an increase in perihematomal cell viability after ICH, suggesting that IRP2 may be a novel target for ICH treatment (Chen et al., 2010). Analysis of the Gene Expression Omnibus (GEO) database (GSE24265) has showed that IRP2 expression is highly expressed in the perihematomal tissues of patients with ICH. Therefore, we speculated that IRP2 may be involved in the regulation of ferroptosis in neuronal cells after ICH.

At present, cell-based therapies have been used to treat stroke. The therapeutic effects of such cell-based therapy are mediated by exosomes released from the administered cells (Zhang et al., 2019). Exosomes are nano-sized vesicles (∼30–200 nm) with a lipid bilayer. Exosomes participate in regulating the intercellular communication through transmitting the intracellular cargos, including RNAs, proteins, metabolites and other substances (Nakase et al., 2017). Increasing studies have indicated that the exosomal miRNA cargo is largely responsible for the therapeutic effects of these exosomes for stroke (Deng et al., 2019; Zhang et al., 2019). In recent years, the crucial role of exosomes derived from miRNA-modified adipose-derived stem cells (ADSCs) in various diseases has been gradually revealed (Geng et al., 2019; Pan et al., 2019; Ren et al., 2019).

miRNAs are endogenous, small (∼22 nucleotides), non-coding RNAs that negatively regulate gene expression by inhibiting mRNA translation or promoting mRNA degradation (Djuranovic et al., 2012). MiR-19b-3p is implicated in several types of cancers and neurodegeneration diseases (Jiang et al., 2017; Wu et al., 2017; Song et al., 2019). MiR-19b-3p is abnormal expressed in embolic stroke and thrombotic stroke (Chen and Jiang, 2018). Whether miR-19b-3p plays a role in ICH has not been reported yet. Analysis of the GEO database (GSE43618) has showed that plasma level of miR-19b-3p is notably decreased in patients with ICH. Our bioinformatics analysis revealed that the ferroptosis-related IRP2 was a putative target of miR-19b-3p. Thus, we speculated that miR-19b-3p may interact with IRP2 to participate in regulating ICH development.

Therefore, in the current study, we evaluated both *in vivo* and *in vitro*, the therapeutic effects of exosomes released from miR-19b-3p-modified ADSCs on ICH- or hemin- induced neurologic injury. We therefore further characterized the interaction between miR-19b-3p and IRP2 and investigated their effects on ferroptosis in ICH.

## Materials and Methods

### Animal Preparation

C57BL/6 mice (8–12 weeks of age) were housed under identical conditions (room temperature at 25°C, 12 h light-dark cycle) and allowed free access to food and water. All experimental procedures were approved by the Ethics of Animal Experiments Committee of the Second Xiangya Hospital of Central South University.

### Collagenase-Induced Mouse Model of ICH and Exosome Administration

Striatal infusion of collagenase was used to induce ICH in mice as previously described (Karuppagounder et al., 2018). Briefly, 1 μl of collagenase (0.075 IU; Sigma-Aldrich, St. Louis, MO) was infused into the right striatum at a flow rate of 0.120 μl/min. In sham-operated animals, 1 μl of saline was infused.

The mice in the ICH+ADSCs-MNC Exos group were administered exosomes (100 μl, 200 μg/ml) secreted by mimic NC-transfected ADSCs through the tail vein at 2 h after ICH. The mice in the ICH+ADSCs-19bM-Exos were administered exosomes (100 μl, 200 μg/ml) from miR-19b-3p mimic-transfected ADSCs through the tail vein at 2 h after ICH.

### Behavioral Tests

Neurological function was evaluated in mice by a modified Neurological Severity Scores (mNSS). mNSS is a comprehensive test for evaluating motor, sensory, balance and reflex abilities. Neurological deficits were graded on a scale of 0–18. Higher score indicated more severe injury (Mao et al., 2013).

### Primary Cortical Neuronal Cultures

The murine primary cortical neurons were isolated from postnatal Day 0 C57BL/6 mice. Briefly, the bilateral cerebral cortex was isolated, minced, and digested in 0.05% trypsin. Cells were plated in six-well plates coated with poly-L-lysine (Sigma-Aldrich) and maintained in Neurobasal medium supplemented with 2% B27, 1% L-glutamine, and 1% penicillin/streptomycin at 37°C with 5% CO_2_. The media were changed every 3 days.

### ADSCs Culture

ADSCs were purchased from BeNa Culture Collection (Beijing, China) and cultured in RPMI-1640 medium containing 10% fetal bovine serum (FBS; Gibco, Thermo Fisher Scientific, Inc., Waltham, MA, United States) and 1% penicillin/streptomycin at 37°C with 5% CO_2_. The media were changed every 3 days. Cells were passaged when they were 90% confluent and were used at the third passage.

### *In vitro* ICH Model and Experimental Design

Hemin was used to model ferroptosis and hemorrhagic stroke in cultured neurons as previously described (Karuppagounder et al., 2018). Cells were treated with hemin (100 μM; Sigma-Aldrich) for 24 h. Cells in the control group were treated with saline. To clarify the regulatory effect of IRP2 on ferroptosis *in vitro*, cells were divided into 3 groups: Hemin, Hemin+sh-NC, and Hemin+sh-IRP2. Briefly, cells were transfected with sh-IRP2 or its negative control sh-NC, and then stimulated with hemin (100 μM) for 24 h. Cell viability, iron content, expression of IRP2, FPN, and TfR1, and contents of malonaldehyde (MDA), glutathione (GSH), glutathione peroxidase 4 (GPX4), and GPX4 protein expression were examined.

### Cell Infection or Transfection

Lentivirus vectors expressing IRP2 shRNA (LV-sh-IRP2) were constructed using Mission Lentiviral Transduction Particles (Sigma-Aldrich) and lentivirus vectors carrying null shRNA were also packaged and used as negative control. The cortical neurons were infected with lentivirus vectors at a multiplicity of infection of 5 using Lipofectamine 3000 reagent (Invitrogen, Thermo Fisher Scientific, Inc.).

The miR-19b-3p mimic and mimic NC were purchased from GenePharma and transfected into the ADSCs using Lipofectamine RNAiMAX reagent (Invitrogen).

### Isolation and Identification of Exosomes

For isolation of exosomes from ADSCs (ADSCs-Exos), ADSCs (control, mimic NC, or the miR-19b-3p overexpression group) at 80% confluency were rinsed with PBS twice and cultured in FBS-depleted RPMI-140 medium for 48 h. The supernatant was collected and centrifuged at 300 × *g* for 20 min to remove dead cells and again at 2,000 × *g* for 20 min to remove cellular debris. The supernatant was filtered with a 0.22-μm filter (Millipore, Billerica, MA, United States) and was used as the conditioned medium of ADSC. Afterward, the ADSCs-derived conditioned medium was centrifugation at 10,000 × *g* for 30 min, and again at 100,000 × *g* for 120 min. The exosome pellets were washed with PBS for three times, filtered with a 0.22 μm filter, and stored at −80°C. All centrifugations were performed at 4°C. The concentrations of exosome proteins were assessed using the BCA Protein Assay Kit (Beyotime, Shanghai, China).

For identification of exosomes, the morphologic characteristics of ADSC-Exos were observed by transmission electron microscopy (TEM). Briefly, the exosome suspension in PBS was adjusted to 500 μg/ml, and fixed in glutaric acid. A 20 μl solution of exosomes was placed on copper grids, post-negatively stained with 3% phosphotungstic acid solution (pH6.8) for 5 min and dried under infrared light. After that, the morphology of the exosomes was observed by TEM. The size distribution of exosomes was evaluated using nanoparticle tracking analysis on a NanoSight NS500 (Malvern Instruments, Malvern, United Kingdom). The protein levels of exosomal surface markers (CD9 and CD63) were examined by western blot.

### Exosome Uptake

The exosomes extracted from the conditioned medium of ADSCs at passage 3 were labeled with 1 μM lipophilic membrane dye DiO (Invitrogen) as previously described (Liang et al., 2016). Then the exosomes were co-cultured with the Dil (Invitrogen)-labeled neurons. The fluorescence distribution and intensity were observed by laser confocal microscopy.

### Cell Viability Assay

Briefly, cortical neurons were seeded in 96-well plates. After the required intervention, MTT (5 mg/ml) was added to each well. After incubation for 4 h, the media were replaced with 150 μl DMSO. The optical density (OD) value was measured at 490 nm by a microplate reader.

### Evaluation of Ferroptosis

The iron deposition, iron content, and levels of MDA, GSH and GPX4 in mouse brains or cortical neurons were measured to evaluate the ferroptosis. The iron deposition in the brain was determined by Perls’ Prussian blue staining using the Perls’ stain Kit (Nanjing SenBeiJia Biological Technology, Nanjing, China) according to the manufacturer’s instructions. The iron content in mouse brains or cortical neurons was measured using the tissue iron assay kit (Nanjing Jiancheng, Nanjing, China) and the iron assay kit (Wuhan AmyJet Scientific, Wuhan, China), respectively. The contents of MDA, GSH, and GPX4 were measured using the MDA ELISA Kit (Nanjing Jiancheng, Nanjing, China), GSH ELISA Kit (Nanjing Jiancheng), and GPX4 ELISA Kit (Shanghai Enzyme Biotechnology, Shanghai, China), respectively.

### Luciferase Reporter Assay

A dual-luciferase reporter assay was performed to validate the interaction between miR-19b-3p and the 3′-UTR of IRP2 mRNA as previously described (Wu et al., 2019). The fragments of the 3′-UTR of IRP2 containing the miR-19b-3p binding site or mutant miR-19b-3p binding sites were amplified using PCR. Then the PCR products were purified and cloned into the pmirGLO vector, namely, IRP2-WT or IRP2-Mut, respectively. HEK293T cells (2 × 10^4^ cells per well) were cultured in 24-well plates for 24 h and then co-transfected with IRP2-WT or IRP2-Mut luciferase reporter plasmids and mimic NC or miR-19b-3p mimic. The luciferase activity was conducted after 48 h of transfection using the luciferase assay kit (Promega, Madison, WI, United States).

### Quantitative Real-Time PCR (qRT-PCR)

Total plasma RNA was extracted from plasma samples using the miRNeasy Serum/Plasma Kit (Qiagen, Germany). Total RNA from brain tissues or cells was extracted using Trizol reagent (Invitrogen). After reverse transcription, whereas the expression levels of miR-19b-3p were using the miRNA qRT-PCR kit (GeneCopoeia, Rockville, MD, United States), whereas the expression of IRP2 was detected using the SYBR premix (Takara, Dalian, Japan) in Applied Biosystems 7500 PCR system. Primers were synthesized by Sangon Biotechnology (Shanghai, China). For internal control, cel-miR-39 was used to determine the standardized expression of miR-19b-3p in plasma samples, while U6 was used for the detection of miR-19b-3p in brain tissues or cells. GAPDH was used as the reference gene for IRP2. Quantification was performed by the 2^–ΔΔ*Ct*^ method.

### Western Blot

The whole-cell lysates were extracted from brain tissues or cells using the radioimmunoprecipitation assay lysis buffer (Beyotime). Equal amounts of protein (30 μg) were resolved by SDS-PAGE and then electrophoretically transferred onto PVDF membranes. The membranes were then blocked with 5% fat-free milk. After overnight incubation at 4°C with the primary antibodies, the membranes were incubated with the horseradish peroxidase-secondary antibodies (dilution: 1:2,000). An enhanced chemiluminescence kit (Pierce; Thermo Fisher Scientific, Inc.) was used to develop blots. The primary antibodies were as follows: anti-IRP2 (1:1,000; Santa Cruz Biotechnology, Dallas, TX, United States), anti- ferroportin (FPN) (1:1,000; Novus Biologicals, Littleton, CO, United States), anti-transferrin receptor (TfR1) (1:1,000; Abcam, Cambridge, MA, United States), and anti-GPX4 (1:1,000; Abcam).

### Statistical Analysis

The unpaired Student’s *t*-test and one-way analysis of variance were used to analyze differences between two or more groups, respectively. All statistical analyses were performed using SPSS version 25.0 (IBM, Chicago, IL, United States). Differences are considered statistically significant at P < 0.05.

## Results

### Decreased miR-19b-3p Expression in ICH Mice

**FIGURE 1:**
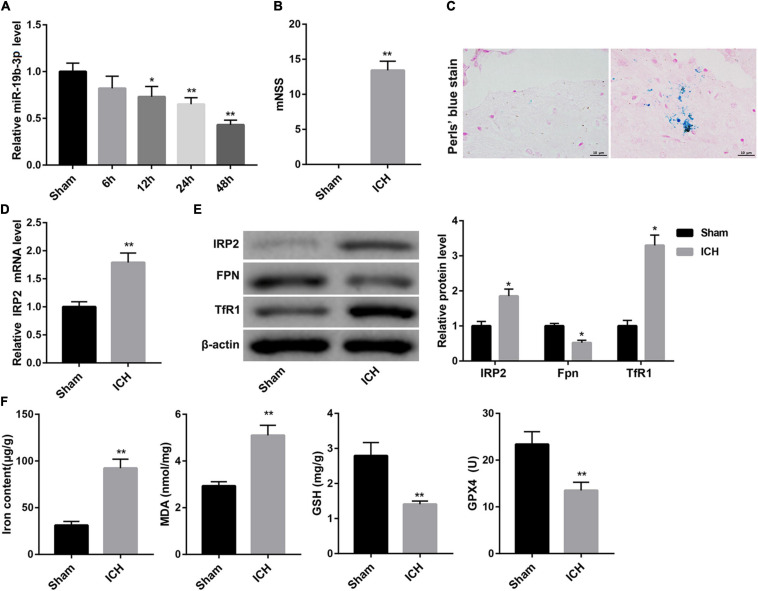
Decreased miR-19b-3p expression and increased ferroptosis in ICH mice. **(A)** Plasma level of miR-19b-3p in sham-operated mice and ICH model mice at 6, 12, 24, and 48 h after ICH was examined by qRT-PCR. **(B)** Neurological function at 48 h after ICH in mice was evaluated by mNSS. **(C)** The iron deposition in the brain at 48 h was determined by Perls’ Prussian blue staining (scale bar: 10 μm). **(D)** qRT-PCR analysis of IRP2 mRNA level and **(E)** western blot analysis of IRP2, FPN, and TfR1 in the brain at 48 h after ICH in mice. **(F)** The contents of iron, MDA, GSH, and GPX4 in the brain at 48 h after ICH in mice. *N* = 6 in each group. *P < 0.05, **P < 0.01, vs. Sham.

Data from qRT-PCR analysis showed that compared with the sham group, miR-19b-3p expression was significantly decreased in the ICH group and became increasingly significant with elapsed time following ICH (Figure 1A). Then we evaluated neurological function by mNSS. A robust increase of mNSS was observed in the ICH group, indicating the more severe neurological injury after ICH with respect to the sham group (Figure 1B).

### Ferroptosis Occurred in ICH Mice

Ferroptosis is a form of iron-dependent cell death that is characterized by the decrease of activity of the lipid repair enzyme GPX4 and GSH and subsequent accumulation of lipid oxidation products, such as MDA (Yang and Stockwell, 2016; Gaschler et al., 2018). Accordingly, we evaluated ferroptosis in the mouse brain by detecting iron deposition, iron content, and levels of MDA, GSH, GPX4, and the protein expression of GPX4. Perls’ Prussian blue staining of brain tissues showed that ICH group exhibited significant increase of iron deposition (Figure 1C). Consistently, the contents of iron and MDA in the brain were greatly higher in the ICH group than that in the sham group. In contrast, the levels of GSH and GPX4, and GPX4 protein expression in the brain were notably decreased in the ICH group (Figure 1F and Supplementary Figure 1A).

Increased iron retention driven by increased TfR1 (an iron import protein) and reduced FPN (an iron export protein) is consistent with increased activity of IRP2 (Deng et al., 2017), a master regulator of cellular iron homeostasis. Data obtained from qRT-PCR and WB revealed that the ICH mice displayed significantly higher mRNA and protein levels of IRP2 in the brain, accompanied by higher protein level of TfR1 and lower protein level of FPN (Figures 1D,E).

### IRP2 Silencing Attenuated Hemin-Induced Ferroptosis in Primary Neurons *in vitro*

**FIGURE 2:**
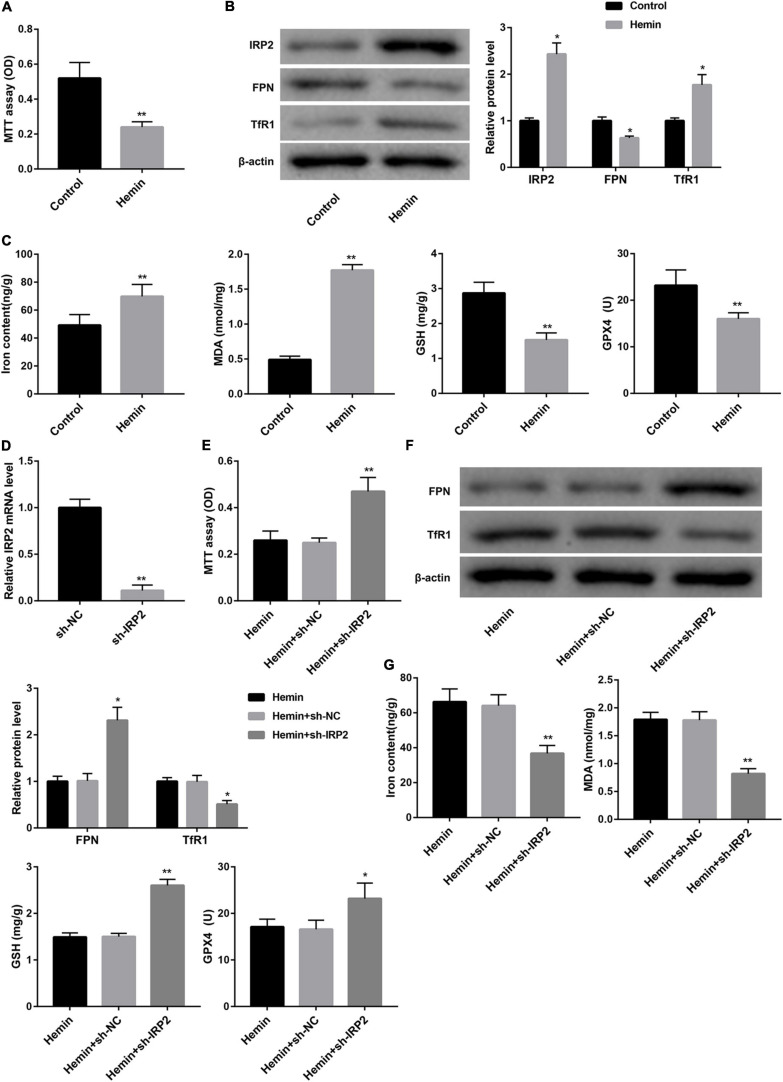
IRP2 silencing attenuated hemin-induced ferroptosis in primary neurons *in vitro*. **(A)** Cell viability examined MTT assay, **(B)** western blot analysis of IRP2, TfR1, and FPN, and **(C)** levels of iron, MDA, GSH, and GPX4 in the primary cortical neurons which were treated with hemin (100 μM) or saline for 24 h. **(D)** Relative IRP2 mRNA level determined by qRT-PCR, **(E)** cell viability examined MTT assay, **(F)** western blot analysis of TfR1 and FPN, and **(G)** levels of iron, MDA, GSH, and GPX4 in the primary cortical neurons which were transfected with sh-IRP2 or sh-NC and treated with hemin (100 μM) for 24 h. The data are presented as the mean ± standard deviation (*n* = 3). *P < 0.05, **P < 0.01, vs. Control or Hemin+sh-NC.

We used hemin to induce ferroptosis and hemorrhagic stroke in cultured neurons. Treatment with hemin significantly repressed cell viability of neurons (Figure 2A). The expression of IRP2 and TfR1 was increased, FPN protein was down-regulated in neurons following hemin treatment (Figure 2B). Furthermore, hemin treatment notably increased levels of iron and MDA, whereas distinctly decreased the levels of GSH and GPX4 and GPX4 protein expression (Figure 2C and Supplementary Figure 1B), suggesting that hemin induced ferroptosis. To examine the effect of IRP2 on hemin-induced ferroptosis, we silenced IRP2 in primary neurons followed by hemin treatment. IRP2 expression was significantly decreased in the primary neurons in the presence of sh-IRP2 (Figure 2D). Importantly, the hemin-induced cytotoxicity (Figure 2E), upregulation of TfR1 (Figure 2F) and ferroptosis (Figure 2G and Supplementary Figure 1C) as well as downregulated of FPN (Figure 2F) were abrogated by IRP2 silencing.

### Identification of ADSCs-Exos

**FIGURE 3:**
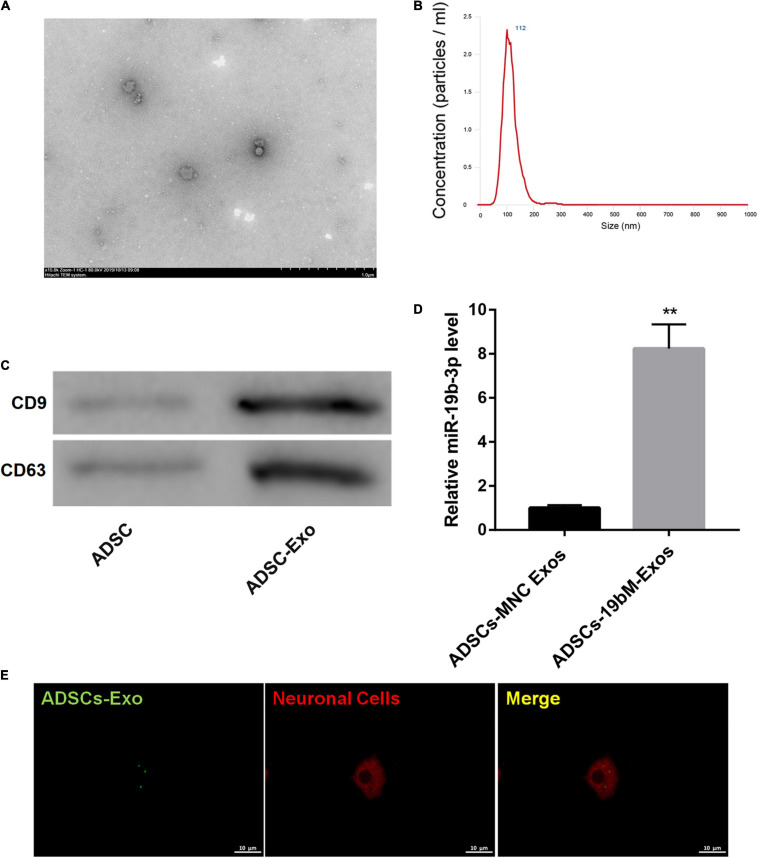
Identification of ADSCs-Exos. **(A)** The morphologic characteristics of ADSCs-Exos were observed by TEM. **(B)** The size distribution of exosomes was evaluated using nanoparticle tracking analysis. **(C)** The protein levels of exosomal surface markers (CD9 and CD63) were examined by western blot. **(D)** miR-19b-3p level in ADSCs-MNC-Exos and ADSCs-19bM-Exos was examined by qRT-PCR. **(E)** The uptake of DiO-labeled ADSCs-Exos (green) by Dil-labeled neuronal cells (red) was observed by laser confocal microscopy. The data are presented as the mean ± standard deviation (*n* = 3). **P < 0.01, vs. ADSCs-MNC-Exos.

TEM showed that ADSCs-Exos were elliptical nanovesicles (Figure 3A). Nanoparticle tracking analysis revealed that ADSCs-Exos exhibited a size distribution between 30 and 200 nm in diameter (Figure 3B). Additionally, these harvested particles displayed positive for exosomal surface markers CD9 and CD63 (Figure 3C). Thus, these data confirmed that these particles isolated from ADSCs were exosomes.

### Exosomal miR-19b-3p Derived From ADSCs Abrogated Hemin-Induced Ferroptosis

To elucidate whether ADSCs-Exos containing high amounts of miR-19b-3p regulates hemin-induced ferroptosis in primary neurons *in vitro*, miR-19b-3p was overexpressed in ADSCs. As shown in Supplementary Figure 2, miR-19b-3p was highly expressed in ADSCs following transfection of miR-19b-3p mimic. Then, primary neurons were co-cultured with exosomes from ADSCs which were transfected with miR-19b-3p mimic or mimic NC (ADSCs-19bM-Exos or ADSCs-MNC-Exos) following hemin treatment. Compared with ADSCs-MNC-Exos, ADSCs-19bM-Exos displayed an up-regulation of miR-19b-3p (Figure 3D). Fluorescence microscopy further confirmed that the DiO (green)-labeled ADSCs-Exo had been taken up and transferred to Dil (red)-labeled neuronal cells (Figure 3E).

**FIGURE 4:**
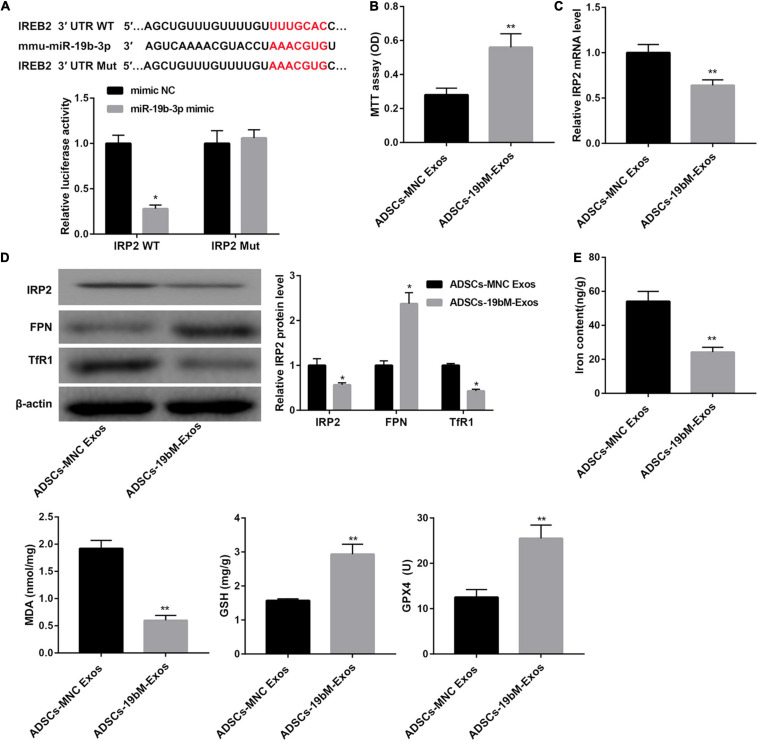
Exosomal miR-19b-3p derived from ADSCs abrogated hemin-induced ferroptosis. **(A)** The putative miR-19b-3p binding sites in IRP2 (IRP2-WT) or and the designed mutant sequence (IRP2-Mut) were indicated. Luciferase reporter assay was conducted to evaluate the interaction ability between IRP2 3′-UTR and miR-19b-3p. **(B)** Cell viability examined MTT assay. **(C)** qRT-PCR analysis of IRP2 mRNA level, **(D)** western blot analysis of IRP2, TfR1, and FPN, and **(E)** levels of iron, MDA, GSH, and GPX4 in the primary cortical neurons which were co-cultured with ADSCs-MNC-Exos or ADSCs-19bM-Exos and hemin (100 μM) for 24 h. The data are presented as the mean ± standard deviation (*n* = 3). *P < 0.05, **P < 0.01, vs. mimic NC or ADSCs-MNC-Exos.

TargetScan analysis^1^ revealed that there were binding sites between miR-19b-3p and RP2 3′-UTR (Figure 4A). In turn, a dual-luciferase reporter assay was performed to verify whether IRP2 was a direct target of miR-19b-3p. Data showed that miR-19b-3p suppressed the luciferase activity of the IRP2 3′-UTR-WT construct, but not the IRP2 3′-UTR-Mut construct in HEK293T cells (Figure 4A), indicating that miR-19b-3p directly targeted IRP2.

Furthermore, when primary neurons were incubated with ADSCs-19bM-Exos under hemin stimulation, cell viability was notably rescued (Figure 4B), IRP2 mRNA and protein levels as well as TfR1 protein level were significantly decreased (Figures 4C,D), whereas FPN protein level was markedly increased (Figure 4D). Moreover, ADSCs-19bM-Exos led to a notable decrease in levels of iron and MDA and a distinct increase in levels of GSH and GPX4 and the expression of GPX4 protein (Figure 4E and Supplementary Figure 1D), which implied that exosomal miR-19b-3p derived from ADSCs abrogated hemin-induced ferroptosis.

### Exosomal miR-19b-3p Derived From ADSCs Inhibited Ferroptosis in ICH Mice

**FIGURE 5:**
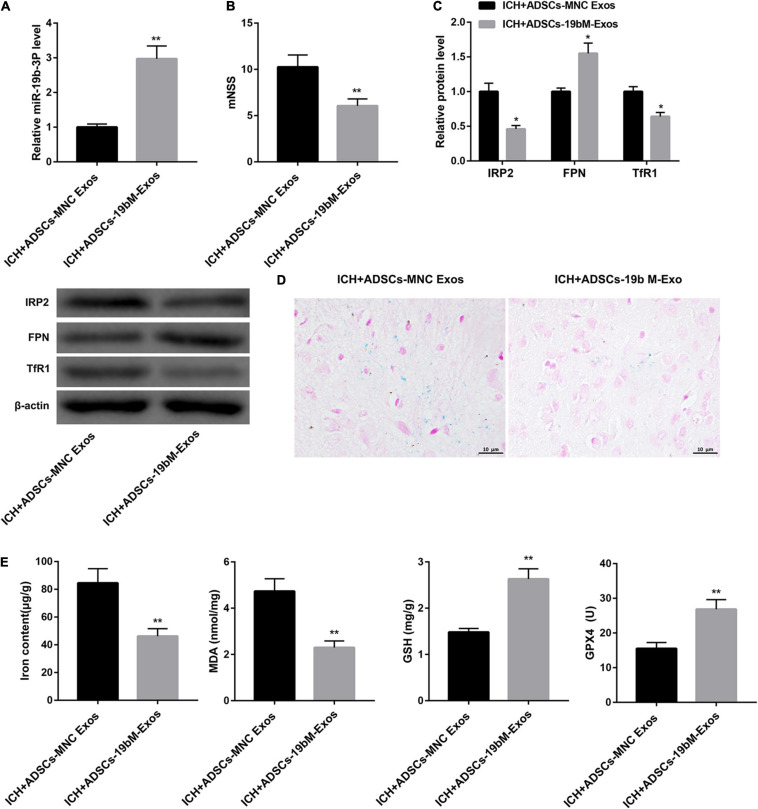
Exosomal miR-19b-3p derived from ADSCs inhibited ferroptosis in ICH mice. Mice were randomly divided into 2 groups: ICH+ADSCs-MNC Exos and ICH+ADSCs-19bM-Exos. **(A)** qRT-PCR analysis of miR-19b-3p level in the plasma at 48 h after ICH in mice. **(B)** Neurological function at 48 h after ICH in mice was evaluated by mNSS. **(C)** Western blot analysis of IRP2, FPN, and TfR1 in the brain at 48 h after ICH in mice. **(D)** The iron deposition in the mouse brain at 48 h was determined by Perls’ Prussian blue staining (scale bar: 10 μm). **(E)** The contents of iron, MDA, GSH, and GPX4 in the brain at 48 h after ICH in mice. *N* = 10 in each group. *P < 0.05, **P < 0.01, vs. ICH+ADSCs-MNC Exos.

To elucidate the biological role of ADSCs-Exos containing high amounts of miR-19b-3p in ICH mice, the animals were randomly divided into 2 groups: ICH+ADSCs-MNC Exos and ICH+ADSCs-19bM-Exos. Plasma level of miR-19b-3p was greatly upregulated in the ICH mice administered ADSCs-19bM-Exos with respect to those mice administered ADSCs-MNC Exos (Figure 5A). Additionally, mice treated with ADSCs-19bM-Exos after ICH exhibited an improved neurologic function, as evidenced by notably decreased mNSS (Figure 5B). The protein levels of IRP2 and TfR1 were significantly decreased, whereas FPN protein was increased in the ICH+ADSCs-19bM-Exos group as compared with the ICH+ADSCs-MNC Exos group (Figure 5C). Perls’ Prussian blue staining of brain tissues showed significantly reduced iron deposition in the ICH+ADSCs-19bM-Exos (Figure 5D). Consistently, the contents of iron and MDA were notably dowregulated, whereas the levels of GSH and GPX4 and the expression of GPX4 protein were greatly upregulated in the brain from ICH mice administered ADSCs-19bM-Exos (Figure 5E and Supplementary Figure 1E). Collectively, these results suggested that ADSCs-Exos-mediated transfer of miR-19b-3p inhibited ferroptosis in ICH mice.

## Discussion

Ferroptosis is initially described by Dixon and colleagues as a unique type of cell death induced by a synthetic compound, erastin (Dixon et al., 2012). When ferroptosis occurs, intracellular iron-dependent lipid peroxide accumulation, reduced GSH and GPX4 expression are observed (Yang and Stockwell, 2016; Gaschler et al., 2018). Furthermore, the increase of intracellular iron content may be the key factor to induce the accumulation of lipid peroxides. Iron is essential for the execution of ferroptosis, which can be reduced by the iron chelator (Dixon et al., 2012; Xie et al., 2016). In the present study, we found that ferroptosis occurred in ICH mice and hemin-treated primary neurons, as evidenced by an increase of iron content and MDA (a product of lipid peroxidation) expression as well as a decrease of GSH and GPX4 expression. Our data implied the involvement of ferroptosis in ICH. Consistently, Li et al. (2017) have reported that ferroptosis has contributed to neuronal death after ICH, and inhibition of neuronal ferroptosis protects hemorrhagic brain.

IRP2 plays a central role in some tumors by enhancing the accumulation of iron (Wang et al., 2014). Additionally, reduction of IRP2 expression has been shown to limit intracellular iron delivery in neurons (Ripa et al., 2017). Overexpression of IRP2 is associated with increased TfR1 (an iron import protein) and decreased FPN (an iron export protein) (Wang et al., 2014). In accordance with these findings, our results also showed that IRP2 silencing decreased TfR1 and increased FPN in the hemin-treated primary neurons. Our findings here suggested a model in which both increased iron uptake (high TfR1) and decreased iron export (low FPN) contributed to supporting high levels of labile iron in ICH. Our findings further suggested that knockdown of IRP2 affected ferroptosis in ICH.

A previous study has demonstrated that perihematomal cell viability after ICH is increased in IRP2 knockout (IRP2^–/–^) mice but not altered in IRP1^–/–^ mice, indicating that IRP2, rather than IRP1, may be a novel therapeutic target for ICH (Chen et al., 2010). Based on this, our results provided further evidence that IRP2 silencing attenuated hemin-induced cytotoxicity and ferroptosis.

Evidence has revealed that certain miRNAs that can target IRP2 may serve as novel therapeutic targets for reducing ferroptosis and improving neuronal injury. For example, Ripa et al. (2017) have found that miR-29 limits intracellular iron delivery in neurons by directly targeting IRP2 and repressing IRP2 expression. In the current investigation, the downregulation of plasma miR-19b-3p level was concomitant with neuronal injury and ferroptosis in ICH mice, suggesting that miR-19b-3p may be closely associated with the advancement of ICH. miR-19b-3p, a cancer-related miRNA (Jiang et al., 2017; Song et al., 2019), has been also shown to contribute to the cognitive function improvement in rats with Alzheimer’s disease (Wu et al., 2017). Thus, it indicates that miR-19b-3p may have a potential neuroprotective function. In the present study, we found that ADSCs-Exos-mediated transfer of miR-19b-3p improved neurologic function in ICH mice, further suggesting the benefit role of miR-19b-3p in exosome-mediated neuroprotection in ICH. Furthermore, our results showed for the first time that exosomes derived from miR-19b-3p-overexpressing ADSCs attenuated ICH-induced ferroptosis, the underlying mechanism may be related to its targeting of the iron regulatory protein IRP2. Specifically, the decreased miR-19b-3p expression and increased IRP2 expression were observed in ICH mice. IRP2 was confirmed as a direct target of miR-19b-3p using luciferase reporter assay. We also found that ADSCs-exosomes containing high amounts of miR-19b-3p decreased IRP2 expression, both at mRNA and protein levels.

In conclusion, the present study demonstrates that exosomes derived from miR-19b-3p-overexpressing ADSCs attenuate ICH-induced ferroptosis and neurologic injury. Exosome therapy in combination with miR-19b-3p may represent a promising strategy for ICH treatment.

## Data Availability Statement

The datasets presented in this study can be found in online repositories. The names of the repository/repositories and accession number(s) can be found below: https://www.ncbi.nlm.nih.gov/geo/, GSE24265 and https://www.ncbi.nlm.nih.gov/geo/, GSE43618.

## Ethics Statement

The animal study was reviewed and approved by The Second Xiangya Hospital of Central South University.

## Author Contributions

XY participated in the design of the project, completed the experiments, and drafted and revised the manuscript. XT instructed the study and revised the manuscript. Both authors read and approved the final manuscript.

## Conflict of Interest

The authors declare that the research was conducted in the absence of any commercial or financial relationships that could be construed as a potential conflict of interest.

## Footnotes

1 http://www.targetscan.org

## Supplementary Material

Supplementary Figure 1 The expression of GPX4 in ICH mice and hemin-treated primary cortical neurons. **(A)** Western blot analysis of GPX4 in the brain at 48 h after ICH in mice. **(B)** Western blot analysis of GPX4 in the primary cortical neurons which were treated with hemin (100 μM) or saline for 24 h. **(C)** western blot analysis of GPX4 in the primary cortical neurons which were transfected with sh-IRP2 or sh-NC and treated with hemin (100 μM) for 24 h. **(D)** western blot analysis of GPX4 in the primary cortical neurons which were co-cultured with ADSCs-MNC-Exos or ADSCs-19bM-Exos and hemin (100 μM) for 24 h. **(E)** Western blot analysis of GPX4 in the brain at 48 h after ICH in mice of ICH+ADSCs-MNC Exos and ICH+ADSCs-19bM-Exos groups. The data are presented as the mean ± standard deviation (*n* = 3). ***P* < 0.01, vs. Sham; ^##^*P* < 0.01, vs. Control; ^&&^*P* < 0.01, vs. Hemin+sh-NC; ^$$^*P* < 0.01, vs. ADSCs-MNC Exos; ^@@^*P* < 0.01, vs. ICH+ADSCs-MNC Exos.

Supplementary Figure 2 Overexpression of miR-19b-3p enhanced miR-19b-3p expression in ADSCs. The qRT-PCR analysis of miR-19b-3p level in ADSCs following transfection of miR-19b-3p mimic or mimic NC. The data are presented as the mean ± standard deviation (*n* = 3). ***P* < 0.01, vs. mimic NC.

## Abbreviations

ICH: intracerebral hemorrhage
IRP1: iron regulatory protein 1
IRP2 (IREB2): iron regulatory protein 2
GEO: Gene Expression Omnibus
ADSCs: adipose-derived stem cells
mNSS: modified Neurological Severity Scores
FBS: fetal bovine serum
TEM: transmission electron microscopy
OD: optical density
MDA: malonaldehyde
GSH: glutathione
GPX4: glutathione peroxidase 4
qRT-PCR: quantitative real-time PCR.
