# Supplementary figures and images for: Exosomes From miR-19b-3p-Modified ADSCs Inhibit Ferroptosis in Intracerebral Hemorrhage Mice
Source: Front Cell Dev Biol. 2021 Jul 7;9:661317. doi: 10.3389/fcell.2021.661317 (PMC8293677; doi:10.3389/fcell.2021.661317)

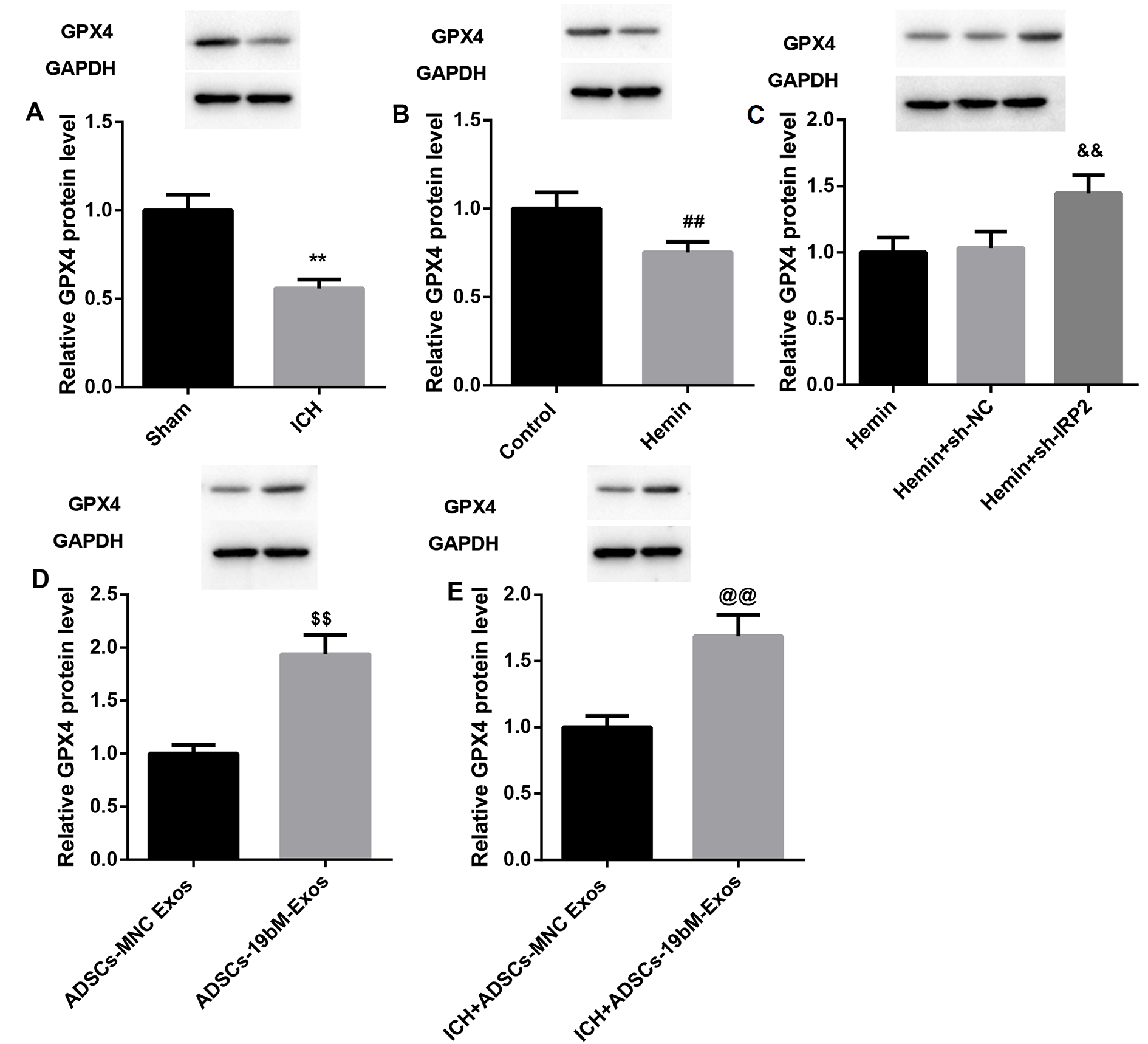

Supplement: Supplementary Figure 1 — The expression of GPX4 in ICH mice and hemin-treated primary cortical neurons. (A) Western blot analysis of GPX4 in the brain at 48 h after ICH in mice. (B) Western blot analysis of GPX4 in the primary cortical neurons which were treated with hemin (100 μM) or saline for 24 h. (C) western blot analysis of GPX4 in the primary cortical neurons which were transfected with sh-IRP2 or sh-NC and treated with hemin (100 μM) for 24 h. (D) western blot analysis of GPX4 in the primary cortical neurons which were co-cultured with ADSCs-MNC-Exos or ADSCs-19bM-Exos and hemin (100 μM) for 24 h. (E) Western blot analysis of GPX4 in the brain at 48 h after ICH in mice of ICH+ADSCs-MNC Exos and ICH+ADSCs-19bM-Exos groups. The data are presented as the mean ± standard deviation (n = 3). **P < 0.01, vs. Sham; ##P < 0.01, vs. Control; &&P < 0.01, vs. Hemin+sh-NC; $$P < 0.01, vs. ADSCs-MNC Exos; @@P < 0.01, vs. ICH+ADSCs-MNC Exos. [file Image_1.TIF]

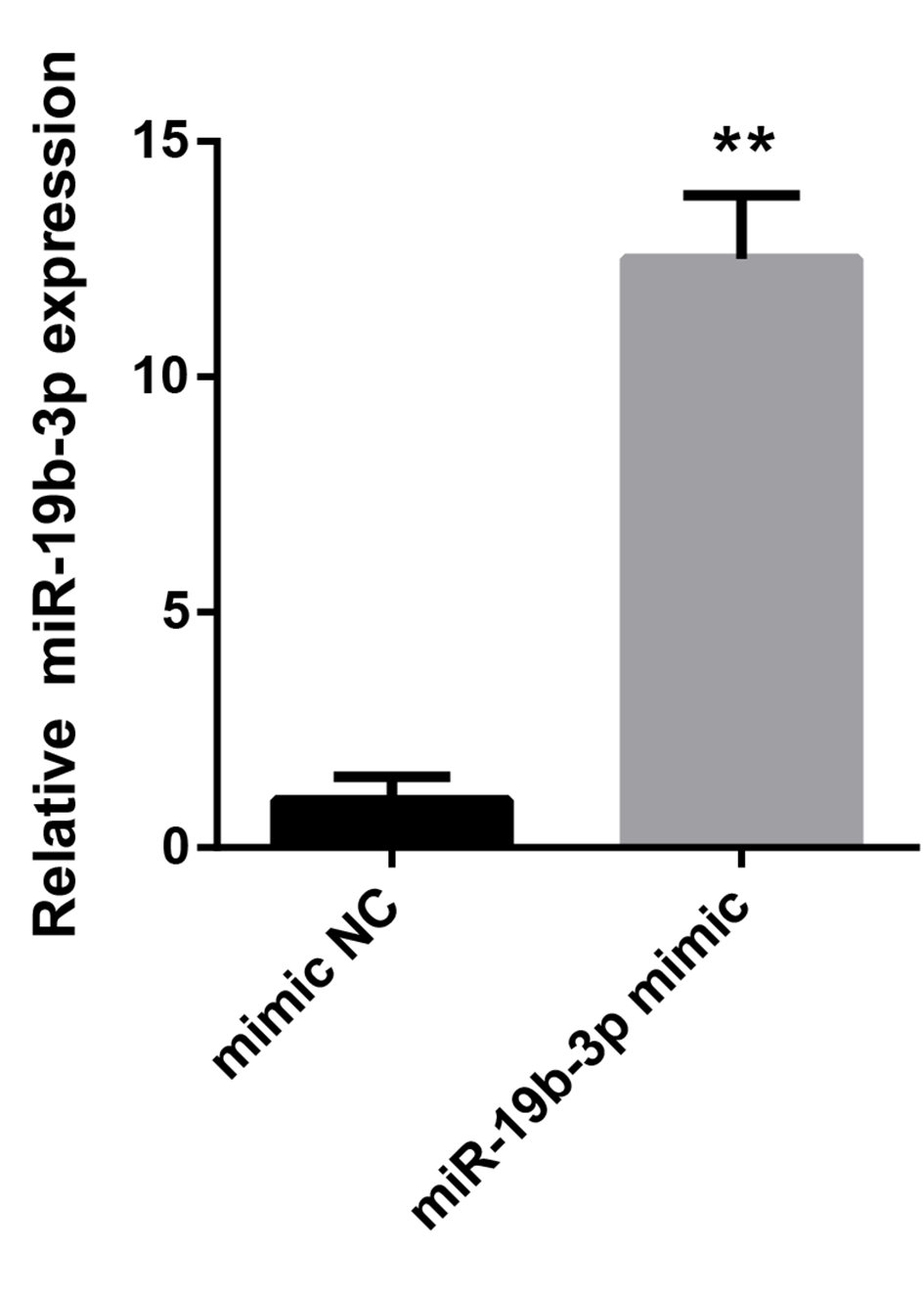

Supplement: Supplementary Figure 2 — Overexpression of miR-19b-3p enhanced miR-19b-3p expression in ADSCs. The qRT-PCR analysis of miR-19b-3p level in ADSCs following transfection of miR-19b-3p mimic or mimic NC. The data are presented as the mean ± standard deviation (n = 3). **P < 0.01, vs. mimic NC. [file Image_2.TIF]
